# An optional choice for closing the duodenal defect after endoscopic full-thickness resection: endoscopic hand-suturing

**DOI:** 10.1055/a-2408-8556

**Published:** 2024-10-14

**Authors:** Yong Liu, Hoi-loi Ng, Shi-Bo Song, Shun He, Gui-Qi Wang

**Affiliations:** 1 Department of Endoscopy, National Cancer Center/National Clinical Research Center for Cancer/Cancer Hospital, Chinese Academy of Medical Sciences and Peking Union Medical College, Beijing, China; 2 Endoscopy Center, Peking University First Hospital, Beijing, China

A 51-year-old man went to hospital for routine examination and a 0-IIa lesion was found in the descending part of the duodenum, which the pathologic result showed to be a neuroendocrine tumor, Grade 1 ( Fig. 1 **a** ). Endoscopic ultrasonography showed the lesion to be located in the muscularis mucosae and submucosal layer, and partly associated with the muscularis propria. After preoperative discussion and consent from the patient, our team decided to perform endoscopic full-thickness resection to remove the lesion ( Fig. 1 **b** ). After resection, endoscopic hand-suturing was used to close the defect uneventfully ( Fig. 1 **c, d, e** , Video 1 ).

**Fig. 1:**
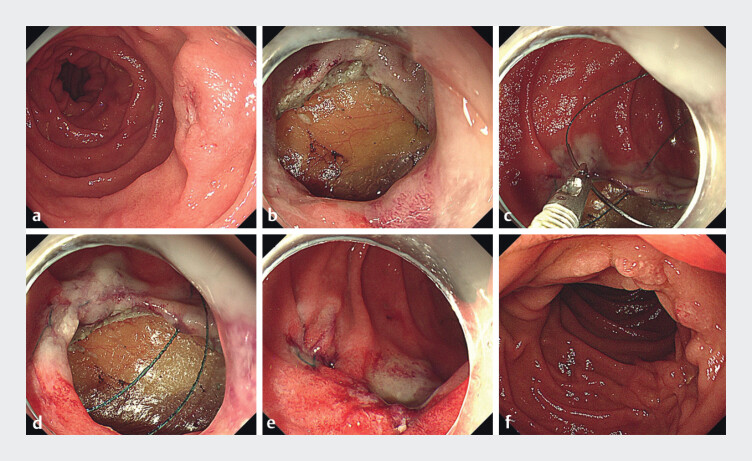
Endoscopic views. **a** 0-IIa lesion located in the descending part of the duodenum. **b** The defect after endoscopic full-thickness resection. **c, d** Endoscopic hand-suturing. **e** The wound after endoscopic hand-suturing. **f** The scar in the descending part of the duodenum after 3 months.

Demonstration of a duodenal defect completely closed using endoscopic hand-suturing after endoscopic full-thickness resection. This method is an option for closing defects located in the duodenum and an alternative to clip closure and laparoscopic suturing.Video 1

The patient was discharged after 7 days, with no bleeding, perforation, or abdominal infection after the procedure. No complications occurred during follow-up, and routine gastroscopy confirmed a good recovery ( Fig. 1 **f** ).

After successful application of endoscopic hand-suturing in gastric and rectal defects 1 2 , our team attempted this technique to suture a duodenal defect. This case confirms the feasibility of endoscopic hand-suturing for closing a defect located in the duodenum, providing an alternative option to clip closure and laparoscopic suturing. (Informed consent was obtained from the patient to publish these images.)

Endoscopy_UCTN_Code_TTT_1AO_2AO

## Notes

Endoscopy E-Videos https://eref.thieme.de/e-videos
*E-Videos* is an open access online section of the journal *Endoscopy* , reporting on interesting cases and new techniques in gastroenterological endoscopy. All papers include a high-quality video and are published with a Creative Commons CC-BY license. Endoscopy E-Videos qualify for HINARI discounts and waivers and eligibility is automatically checked during the submission process. We grant 100% waivers to articles whose corresponding authors are based in Group A countries and 50% waivers to those who are based in Group B countries as classified by Research4Life (see: https://www.research4life.org/access/eligibility/ ). This section has its own submission website at https://mc.manuscriptcentral.com/e-videos .
